# Editorial: Metal Biology Takes Flight: The Study of Metal Homeostasis and Detoxification in Insects

**DOI:** 10.3389/fgene.2018.00221

**Published:** 2018-06-19

**Authors:** Stephanie E. Mohr, David W. Killilea

**Affiliations:** ^1^Harvard Medical School, Boston, MA, United States; ^2^Nutrition and Metabolism Center, Children's Hospital Oakland Research Institute, Oakland, CA, United States

**Keywords:** minerals, toxicity, copper, iron, heme, manganese, molybdenum, mercury

Like all animals, insects must acquire nutrient metals such as copper, iron, manganese, and zinc from their environment or their hosts for essential growth and metabolic functions. When availability of these metals is low, high-affinity metal importers and binding proteins are required to maintain adequate metal intake. Alternatively, when exposure to specific metals is very high, such as iron levels for blood-feeding insects such as mosquitoes, efficient metal exporters and intracellular chelators are needed to prevent toxicity. Furthermore, nutrient metal balance must be achieved while simultaneously minimizing the intake of toxic metals such cadmium, lead, and mercury, necessitating use of selective transporters and stress response systems. All of these processes must be coordinated to maintain the proper balance of metals within insects in the face of variable metal availability.

Humans must also acquire sufficient nutrient metals from their diet while avoiding excess or toxic metals for optimal health. Imbalances in nutrient metals and exposure to toxic metals are both public health concerns since they are linked to numerous pathological conditions, including neurodegenerative disease, cardiovascular dysfunction, and human metabolic disorders (Ayton et al., 2013; Rines and Ardehali, 2013; Dusek et al., 2015; Ferreira and Gahl, 2017). Understanding the pathways and mechanisms of metal imbalances and subsequent toxicity are highly active areas of current biomedical research that have inspired new classes of therapeutics targeting metal metabolism (Barnham and Bush, 2014; Weekley and He, 2017).

Studying metal imbalances in insects provides a number of opportunities since they are diverse, often can be easily maintained in large populations in the lab, and, in the case of *Drosophila*, offer a well-studied model with detailed genetics, a defined life cycle, and a relatively short lifespan. Furthermore, sophisticated genetic tools are available for the study in *Drosophila* of human diseases such as neurodegenerative diseases and genetic disorders that are relevant to metal biology (Calap-Quintana et al., 2017). Moreover, *Drosophila* studies can be complemented by direct study of other insects, including disease vectors such as mosquitoes and kissing bugs in which metal-related cellular activities related to blood feeding can be addressed. These studies can lead to an improved understanding of conserved and species-specific aspects of metal biology. Additionally, this knowledge might provide new opportunities to control insect populations and attenuate insect-borne disease.

This Frontiers Research Topic, *Metal Biology Takes Flight: The Study of Metal Homeostasis and Detoxification in Insects*, brings together a collection of articles contributed by researchers from seven different countries and with different expertise that address key aspects of metal regulation in insects. Studies of several species and from both laboratory and field settings are addressed in the collection, with authors approaching relevant questions from both experimental and theoretical perspectives. In primary research articles, (1) Tsujimoto et al. identify candidate metal transporters that might play a key role in protecting *Aedes aegypti*, the mosquito vector of Zika, chikungunya and other disease-causing viruses, from iron toxification following a bloodmeal, and (2) Walter-Nuno et al. identify key genes that protect the kissing bug *Rhodnius prolixus*, a blood-feeding insect that is a vector of Chagas disease, from blood iron toxicity. Also, (3) Prince and Rand identify genes that protect *Drosophila* from methyl-mercury toxicity. In review articles that focus on *Drosophila*, (4) Navarro and Schneuwly address metal imbalances with connections to Friedreich's Ataxia, Menkes and Wilson's diseases, whereas (5) Xiao and Zhou address metal imbalances with connections to Spondylocheirodysplasia-Ehlers-Danlos Syndrome disease. In a hypothesis paper, (6) Marelja et al. examine the iron-sulfur cluster and molybdenum cofactor regulation and the potential impact on iron and heme metabolism in *Drosophila*. Finally, several reviews compare metal homeostasis in multiple insect species, including (7) Merritt and Bewick, who highlight the genetic and epigenetic mechanisms used to deal with metal toxicities; (8) Whiten et al. detail the different mechanisms used to prevent iron toxicity in blood-sucking arthropods; and (9) Ben-Shahar describes the integration of responses to manganese toxicity.

In our view, insects are relevant and appropriate model systems in which to uncover new information about metal homeostasis and detoxification that will ultimately improve public health. The articles in this collection just scratch the surface of the possibilities of this field. In putting together this Research Topic, we have endeavored to strengthen ties between studies in model and non-model insects, and hope that the reported research, reviews, and hypotheses in this topic will inspire new studies.

## Author contributions

All authors listed have made a substantial, direct and intellectual contribution to the work, and approved it for publication.

### Conflict of interest statement

The authors declare that the research was conducted in the absence of any commercial or financial relationships that could be construed as a potential conflict of interest.
